# Clinicopathologic Features of Isolated AFOP Nodules Radiologically Mimicking Malignancy in Post COVID-19 Patients: A Case Series Study

**DOI:** 10.3390/jcm14113968

**Published:** 2025-06-04

**Authors:** Massimiliano Mancini, Lavinia Bargiacchi, Gisella Guido, Fabiana Messa, Beatrice Trabalza Marinucci, Erino Angelo Rendina, Mohsen Ibrahim, Andrea Vecchione

**Affiliations:** 1 Morphologic and Molecular Pathology Unit, St. Andrea University Hospital, Sapienza University of Rome, 00189 Rome, Italy; 2 Department of Medical and Surgical Sciences and Translational Medicine, Faculty of Medicine and Psychology, Sapienza University of Rome, 00189 Rome, Italy; 3 Radiology Unit, Department of Surgical and Medical Sciences and Translational Medicine, Sant’ Andrea University Hospital, Sapienza University of Rome, Via di Grottarossa 1035-1039, 00189 Rome, Italy; 4 Department of Thoracic Surgery, Sant’ Andrea Hospital, Sapienza University of Rome, 00189 Rome, Italy; 5 Morphologic and Molecular Pathology Unit, Department of Clinical and Molecular Medicine, St. Andrea University Hospital, Sapienza University of Rome, 00189 Rome, Italy

**Keywords:** organizing pneumonia, fibrinous, alveolar damage, COVID-19

## Abstract

**Background/Objectives:** Acute Fibrinous and Organizing Pneumonia (AFOP) is a rare pulmonary condition histologically characterized by intra-alveolar fibrin deposition and organizing pneumonia without hyaline membranes. This study aims to describe the clinicopathologic and radiologic features of isolated AFOP nodules presenting as solitary pulmonary nodules (SPNs) mimicking malignancy in patients with recent COVID-19 infection. **Methods:** We retrospectively analyzed consecutive cases of histologically confirmed AFOP (*n* = 20) and organizing pneumonia (OP; *n* = 119) presenting radiologically as SPNs suspicious for malignancy from January 2021 to December 2023. Clinical data, COVID-19 status, radiologic features (including nodular characteristics, ground-glass opacity [GGO], and consolidation), and histopathological findings were collected and analyzed. Digital image analysis quantified the intra-alveolar fibrin content. **Results:** AFOP nodules showed a significant association with previous COVID-19 infection compared to OP (55% vs. 0.8%, *p* < 0.001). Radiologically, AFOP lesions were predominantly located in the upper lobes, frequently exhibiting a mixed pattern of GGO and consolidation within solitary nodules (8–28 mm diameter), distinctly differing from the predominantly lower-lobe homogeneous consolidations in OP. Histologically, AFOP was defined by prominent intra-alveolar fibrin “balls,” correlating significantly with radiological consolidation patterns (*r* = 0.991, *p* < 0.05). Regions of consolidation demonstrated higher fibrin contents compared to areas of predominant GGO. **Conclusions:** Isolated AFOP nodules presenting as SPNs post-COVID-19 infection strongly mimic malignancy radiologically, highlighting the necessity for multidisciplinary diagnostic approaches integrating radiological and histopathological data to avoid unnecessary interventions. Recognition of this rare but distinctive clinical entity is essential for appropriate patient management.

## 1. Introduction

Acute Fibrinous and Organizing Pneumonia (AFOP) is an uncommon but distinct histopathological entity first described by Beasley et al. in 2002 [1]. It is characterized by the presence of intra-alveolar fibrous plugs encompassing varying amounts of fibrin deposition, often forming “fibrin balls”, which create a “fibrinous” organizing pneumonia pattern. This pattern is notably distinct due to the absence of hyaline membranes, a key feature differentiating it from other forms of diffuse alveolar damage (DAD) and classic organizing pneumonia (OP).

AFOP has been reported across diverse clinical settings, both idiopathic and secondary to infections, autoimmune diseases, drug reactions, and environmental exposures [2,3,4,5]. Despite its well-defined histological characteristics, AFOP remains underrecognized in clinical practice, partially due to its overlapping clinical and radiological features with other forms of interstitial lung disease. Patients commonly present with non-specific symptoms such as cough, dyspnea, fever, and malaise, which pose significant diagnostic challenges. Laboratory findings typically show elevated inflammatory markers, including C-reactive protein (CRP), white blood cell count, and IL-6 levels, but these are non-specific indicators of inflammation [6].

Radiologically, AFOP exhibits a wide range of imaging patterns, including bilateral patchy consolidations, ground-glass opacities, and nodular infiltrates, often with basal predominance. These radiologic features frequently overlap with other interstitial lung diseases, such as OP, infections, and neoplastic processes, making histopathological examination essential for a definitive diagnosis [5,7].

Two distinct clinical variants of AFOP have been identified: an acute fulminant variant and a subacute variant. The acute variant typically progresses rapidly to severe respiratory failure, often necessitating mechanical ventilation and carrying a high mortality rate. In contrast, the subacute variant follows a more indolent course, with relatively better outcomes and less frequent need for mechanical ventilation [8].

Therapeutically, AFOP often requires a combination of antibiotics, corticosteroids, and, in some cases, immunosuppressants such as mycophenolate mofetil, azathioprine, and cyclophosphamide. Long-term corticosteroid therapy has been associated with improved outcomes, particularly in subacute presentations. However, there is no standardized treatment protocol, and therapeutic decisions are largely guided by clinical presentation, underlying etiology, and disease severity [9,10]. Clinical evolution, blood gas, and imaging changes of patients [8] with long-term corticosteroid therapy are associated with better outcomes.

Histologically, AFOP is defined by a distinctive intra-alveolar fibrin deposition forming dense eosinophilic “fibrin balls” within alveolar spaces. This is accompanied by features of organizing pneumonia, including fibroblastic plugs in alveolar ducts and airspaces, composed of fibroblasts and myofibroblasts embedded in loose connective tissue [1]. Importantly, classic hyaline membranes, a hallmark of DAD, are absent in AFOP. The alveolar walls surrounding fibrin deposits often exhibit mild to moderate lymphoplasmacytic infiltrate, type 2 pneumocyte hyperplasia, and variable degrees of interstitial widening. While AFOP shares some overlapping histologic features with both DAD and OP, it remains a distinct entity. Compared to OP, AFOP is more fibrin-dominant, with intra-alveolar fibrin plugs forming the primary histological hallmark, while OP predominantly exhibits fibroblastic Masson bodies [1,11].

Interestingly, AFOP has been observed as a pattern in various infectious and non-infectious etiologies, including SARS-CoV-2 infection, drug-induced lung injury, and hypersensitivity pneumonitis. Recent studies have highlighted its occurrence in patients with severe COVID-19 pneumonia, further emphasizing the importance of recognizing this distinct pattern in histopathological examinations [12].

Despite its clear histological definition, AFOP remains underdiagnosed, and its prognostic significance is not fully understood. Current evidence suggests that mechanical ventilation is a strong predictor of poor outcomes, with nearly all ventilated patients succumbing to the disease [13]. However, the subacute variant, particularly when promptly recognized and treated, often demonstrates favorable long-term outcomes.

This study aims to expand the current understanding of AFOP by describing the clinicopathologic features of a case series, with a particular focus on its nodular presentation. The nodular variant of AFOP is relatively underreported in the literature, and its recognition may have significant implications for diagnosis and management. By providing a comprehensive analysis of AFOP cases, including clinical presentation, imaging findings, histological characteristics, and treatment outcomes, we hope to contribute to improved diagnostic accuracy and therapeutic strategies, ultimately enhancing patient outcomes.

## 2. Materials and Methods

### 2.1. Case Selection

We retrospectively reviewed consecutive cases of solitary pulmonary nodules (SPNs) diagnosed histologically as Acute Fibrinous Organizing Pneumonia and Organizing Pneumonia at our institution during the past three years (from January 2021 to December 2023), presenting radiologically as pulmonary solitary nodules or consolidations mimicking neoplasia and diagnosed as OP and AFOP. All patients underwent conservative surgery (i.e., wedge resection or segmentary resection depending on the dimensions of the lesions) after frozen section examination due to isolated nodules suspicious for malignancy. Inclusion criteria were based on histopathological confirmation of OP and AFOP characterized by intra-alveolar fibrin and organizing pneumonia without hyaline membranes. Clinical data, including patient demographics, presenting symptoms, underlying conditions, and outcomes, were prospectively collected and retrospectively extracted from medical records.

All radiological assessments were performed by a single experienced thoracic radiologist (G.G.) following standardized criteria. Histopathological evaluations were conducted independently by three experienced thoracic pathologists (M.M., L.B., and A.V.). Discrepancies were discussed in joint review sessions, and final diagnoses were reached by consensus to ensure consistency and diagnostic accuracy.

### 2.2. CT Acquisition Technique and CT Image Analysis

All chest high-resolution computed tomography (HRCT) acquisitions were obtained with the patients in supine position during end-inspiration without contrast medium injection. HRCT was performed on a 128-slice CT (GE Revolution EVO 64 Slice CT Scanner, GE Medical Systems, Milwaukee, WI, USA) with the following technical parameters: tube voltage of 120 kV; tube current modulation of 100–250 mAs; spiral pitch factor of 0.98; and collimation width of 0.625. Image reconstructions were made with convolution kernel BONEPLUS at a slice thickness of 1.25 mm.

All the scans were retrospective reviewed by a thoracic radiologist. The following CT features were recorded: the presence, size, attenuation and distribution of nodules/consolidations, eventual surrounding findings, ground-glass opacities (GGOs), and interstitial septal thickening and presence of pleural effusion. Furthermore, correlations between radiological features and histopathological findings were evaluated.

### 2.3. Histopathological Examination

Lung biopsies were processed using standard histological techniques. Hematoxylin and eosin (H&E) staining was employed to identify key histopathological features, including the presence of intra-alveolar fibrin, organizing pneumonia, and other relevant tissue alterations. Special histochemical stains, such as Masson’s trichrome, were utilized to highlight fibrotic components, enabling precise visualization of collagen deposition and fibrin content within the lung parenchyma.

Whole-slide images (WSIs) of Masson’s trichrome-stained sections were digitally acquired for analysis. Images were processed using FIJI software (version 2.16.0/1.54p), where individual color channels were separated. Specifically, the blue channel, corresponding to the fibrin and collagen-rich areas, was isolated and analyzed to quantify fibrin content across regions of interest (ROIs).

## 3. Results

In this study, we analyzed 20 patients with AFOP (Acute Fibrinous Organizing Pneumonia) nodules (cases) and 119 patients with OP (organizing pneumonia) nodules (controls), and the clinical characteristics are summarized in Table 1.

The gender distribution in the AFOP group showed a higher proportion of females, with 14 (70%) females and 6 (30%) males, while the OP group had a more balanced gender ratio, with 56 (47.1%) females and 63 (52.9%) males. The mean age of patients in the AFOP group was 65.85 years (±9.1) compared to 66.83 years (±12.8) in the OP group, with no statistically significant difference in the mean age between the two groups (*p* > 0.05).

Cigarette smoking was more prevalent in the OP group, with 42 (35.3%) smokers compared to 5 (25%) in the AFOP group, although this difference did not reach statistical significance (*p* = 0.38). Cannabinoid use and e-cigarette smoking were rare in both groups, with no significant differences observed.

**Figure 1:**
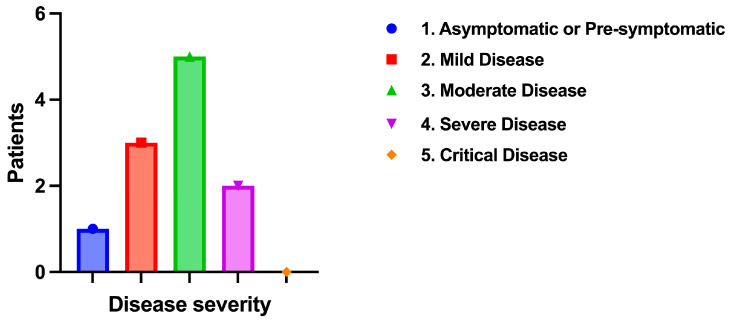
Patients’ distribution according to COVID-19 severity.

A significant finding was the difference in COVID-19 infection rates. Among the AFOP patients, 11 (55%) had a history of COVID-19, while only 1 (0.8%) patient in the OP group had COVID-19. Disease severity was graded according to the World Health Organization (WHO)’s classification of COVID-19 disease [14]. Among the AFOP patients with COVID-19, one patient was asymptomatic, three had mild disease, five had moderate disease, one had severe disease, and none had critical disease (Figure 1).

Notably, COVID-19 infection occurred 90 to 180 days before the CT scan that detected the nodularity, which is usually performed due to persistent cough or pulmonary function deterioration. This difference in COVID-19 prevalence was statistically significant (*p* < 0.001), indicating a potential link between COVID-19 and the development of AFOP nodules. In contrast, 14 (11.8%) patients in the OP group had other pulmonary infections, whereas no cases of other pulmonary infections were observed in the AFOP group, a difference that was statistically significant (*p* < 0.05).

### 3.1. Radiological Characteristics

**Figure 2:**
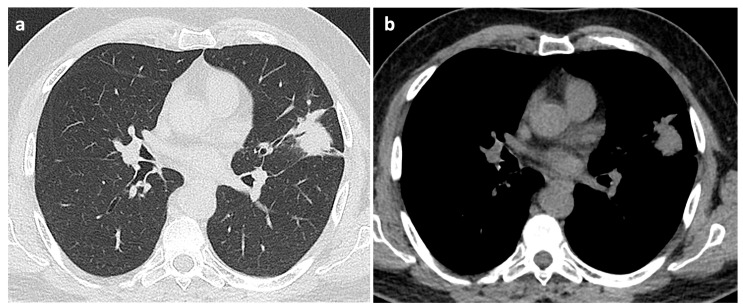
Parenchymal (**a**) and mediastinal (**b**) windows of axial thin-section unenhanced chest CT scans of a 54-year-old man with AFOP. Images show pseudonodular consolidation in the left upper lobe adjacent to the left fissure, with thin air bronchogram and a neighboring micronodule.

High-resolution computed tomography (HRCT) scans revealed nodular opacities in 95% of patients diagnosed with AFOP, predominantly affecting the right upper lobe. This preferential localization might reflect underlying regional factors, such as airflow dynamics or specific anatomical vulnerabilities within the upper pulmonary zones. The AFOP nodules exhibited considerable variability in size, ranging from 8 mm to 28 mm in diameter, indicating heterogeneity in disease progression and inflammatory responses. Radiologically, AFOP commonly presented as GGO nodules or pseudonodular consolidations (Figure 2) (9/20), reflecting alveolar damage and partial alveolar filling, or more frequently as diffuse patchy GGO (11/20) with varying degrees of consolidation, indicative of denser alveolar involvement characterized by fibrin deposition, inflammatory exudates, and cellular infiltrates.

The AFOP nodules often showed surrounding interstitial septal thickening or “tree-in-bud” micronodules (15/20); moreover, they were frequently associated with subpleural bands, typical findings after COVID-19 pneumonia (13/20). This radiological appearance could underscore the dynamic nature of AFOP nodules, highlighting ongoing cycles of fibrin-rich exudate deposition and resolution.

**Figure 3:**
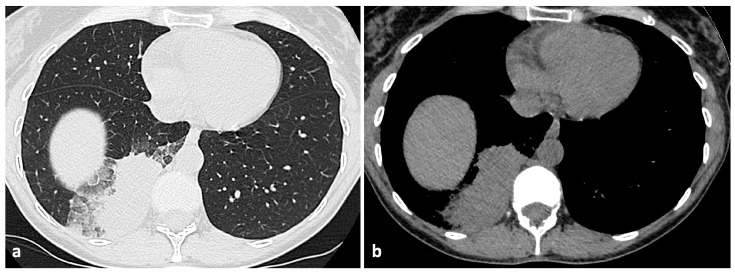
Parenchymal (**a**) and mediastinal (**b**) windows of axial thin-section unenhanced chest CT scans of a 35-year-old woman with OP. Images show a large parenchymal consolidation in the right lower lobe, with subpleural distribution, surrounded by patchy GOO alterations and interstitial septal thickening.

In contrast, nodular opacities associated with OP displayed distinct radiological and anatomical features, despite histopathological similarities with AFOP within the spectrum of organizing lung injury. OP alterations predominantly tended to be more uniform in size and morphology, shown as more homogeneous parenchymal consolidations (Figure 3) with air bronchogram (104/119), and they were less commonly associated with pronounced consolidation seen in AFOP cases, while GGOs were rarely also observed in OP (15/119).

Pleural effusion was detected in some cases of both AFOP (3/19) and OP (11/119). Furthermore, OP consolidations had main peripheral and subpleural distributions, frequently favoring the lower lobes, contrasting with the upper lobe preference observed in AFOP. Additionally, the radiological hallmark of OP—the “reverse halo sign”—characterized by central clearing encircled by a ring of consolidation, was notably absent in our AFOP series, where consolidative changes appeared more diffuse and lacked this specific radiological feature.

### 3.2. Histopathologic Features

**Figure 4:**
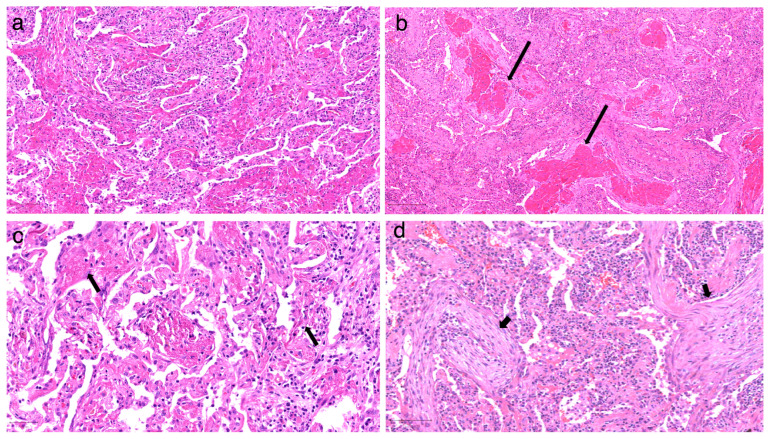
(**a**) The hallmark histopathologic feature of AFOP is the presence of intra-alveolar fibrin balls admixed with organizing pneumonia. (**b**) The long arrow indicates the filling of alveolar spaces with intra-alveolar fibrin balls, characteristic of AFOP. (**c**) Fibrin deposition (short arrow) mimics hyaline membranes typically seen in ARDS; however, AFOP notably lacks true hyaline membranes. (**d**) The organizing pneumonia (OP) pattern shows fibroblastic plugs (arrowheads) of granulation tissue occupying alveolar spaces.

Histologically, AFOP nodules were characterized by prominent intra-alveolar fibrin deposition, forming well-defined fibrin balls that represent the hallmark of this pathological entity (Figure 4).

These fibrin balls appeared as dense, eosinophilic aggregates of fibrin, occasionally interspersed with acute inflammatory cells, indicating ongoing alveolar injury and exudative response. The fibrin deposits frequently presented an irregular shape and varied density, reflecting dynamic phases of fibrin accumulation and partial resolution. Surrounding alveolar spaces exhibited granulation tissue composed of fibroblasts and myofibroblasts embedded within a loose connective tissue matrix, suggestive of early reparative processes following alveolar injury. This granulation tissue often extended into adjacent alveolar ducts, forming fibromyxoid plugs that contributed to the architectural distortion observed in the affected regions.

In patients with a prior history of COVID-19 infection, additional histological patterns influenced AFOP presentation and severity. Notably, seven out of eleven patients with documented prior COVID-19 exhibited varying degrees of diffuse alveolar damage (DAD), characterized by hyaline membrane formation, alveolar septal thickening, and inflammatory cell infiltration—findings consistent with acute and subacute COVID-19-related lung injury. Interestingly, a temporal correlation was noted between the interval from COVID-19 infection to AFOP detection and the fibrin content within alveolar plugs. Shorter intervals correlated with higher intra-alveolar fibrin deposition, reflecting an intensified inflammatory and exudative response in the early post-infective phase.

**Figure 5:**
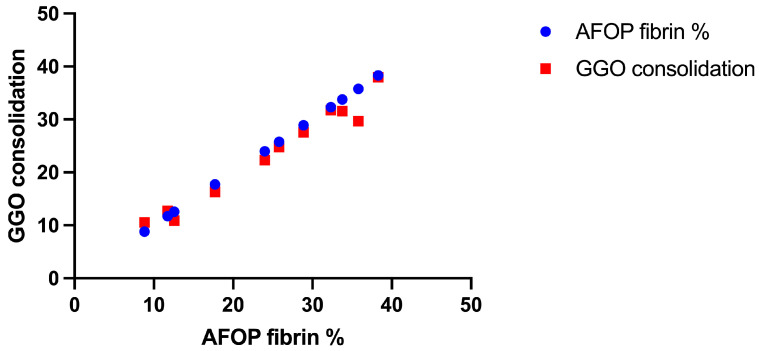
Correlation between histological fibrin deposition and radiological consolidation.

Importantly, intra-alveolar fibrin deposition demonstrated a robust and statistically significant positive correlation (*r* = 0.991, *p* < 0.05; no correction for multiple comparisons was applied) with radiological findings on HRCT scans (Figure 5), underscoring the diagnostic relevance of integrating histological and imaging data.

Areas with prominent intra-alveolar fibrin accumulation were positively associated with ground-glass opacities (GGOs) and regions of consolidation on imaging. This correlation suggests that the fibrin-rich exudates within the alveolar spaces are a major contributor to the radiological appearance of GGOs, where partial filling of alveoli with fibrin and inflammatory debris reduces the air content and increases parenchymal density. Furthermore, regions exhibiting denser fibrin plugs were more likely to correspond to areas of consolidation, where complete alveolar filling resulted in opacified lung zones with sharp delineation from adjacent normal parenchyma.

**Figure 6:**
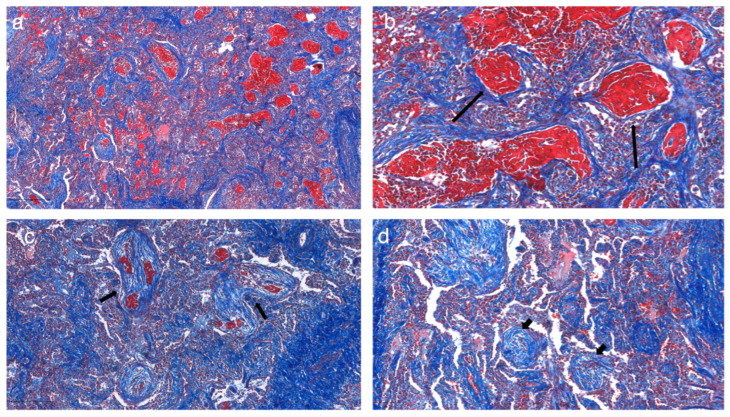
(**a**) Masson’s trichrome staining highlights features of AFOP, providing contrast for the fibrin-rich areas. (**b**) The long arrows indicate fibrin balls, stained red, a defining feature of AFOP. (**c**) Red staining (short arrows) demonstrates fibrin within alveolar spaces, interspersed with granulation tissue, which characterizes the AFOP pattern. (**d**) In contrast, the OP pattern (arrowheads) contains only fibroblasts and myofibroblasts within a loose connective tissue matrix stained blue, indicating a lack of fibrin balls.

These findings underscore the intricate relationship between histological and radiological features in AFOP, providing insights into the dynamic interplay between alveolar injury, fibrin deposition, and the subsequent reparative response. The positive correlation between intra-alveolar fibrin and radiological patterns not only reinforces the diagnostic utility of imaging but also highlights the underlying pathological processes driving disease manifestation. This histo-radiological concordance emphasizes the importance of integrating microscopic findings with imaging data to achieve a more nuanced understanding of disease progression, particularly in patients with prior COVID-19 infection, where the inflammatory milieu may exacerbate fibrin deposition and subsequent radiological changes (Figure 6).

## 4. Discussion

This study investigates the association between Acute Fibrinous and Organizing Pneumonia (AFOP) and COVID-19 infection by comparing it with organizing pneumonia (OP). Our findings indicate a significant association: 55% of AFOP patients had a history of COVID-19 infection compared to only 0.8% in the OP group. This strong link underscores the necessity to consider AFOP as a potential post-COVID pulmonary manifestation.

Histologically, AFOP is characterized by prominent intra-alveolar fibrin deposition, distinguishing it clearly from OP, which typically shows less extensive fibroblastic plugs [15]. This distinct histopathological profile frequently mimics malignancy on imaging, creating substantial diagnostic challenges. Our study further reveals notable differences between AFOP and OP regarding gender distribution and smoking history, suggesting differing underlying pathogenic mechanisms.

AFOP is hypothesized to represent a transitional stage within a spectrum encompassing Diffuse Alveolar Damage (DAD) and OP [1]. Although AFOP can be idiopathic, it commonly occurs secondary to various conditions such as infections, autoimmune diseases, drugs, hematologic malignancies, and inhalation injuries [16]. Recent studies have also identified AFOP as a key histopathological pattern associated with severe lung injury in critically ill COVID-19 patients, characterized by intra-alveolar fibrin deposition and cellular organization contributing to potential lung fibrosis [14,17,18].

Our results highlight a significant difference in infection profiles between the AFOP and OP groups; specifically, while 11.8% of OP patients presented with various pulmonary infections, none were observed in AFOP cases aside from COVID-19. This emphasizes AFOP’s association with severe viral insults, particularly COVID-19, in contrast to OP, which may relate to milder or subacute infectious etiologies [19,20,21].

Interestingly, our data reveal a predominance of females (70%) among AFOP patients, contrasting previous studies in the literature that suggest male predominance [1,2]. This discrepancy warrants further investigation to explore potential gender-specific immunological or pathophysiological responses related to COVID-19 infection [22]. Additionally, although smoking prevalence differed between groups (35.3% in OP vs. 25% in AFOP), our findings did not indicate a statistically significant role for smoking in AFOP pathogenesis [23].

It is worth noting that in our study, the majority of AFOP cases were detected in patients affected by mild and moderate COVID-19 pneumonia; as stated in an interesting case series study and literature review conducted by Kim JY et al. [24], the clinical course and radiological findings of AFOP reflect one another. In this perspective, according to our study, patients with severe progressive course showed CT findings similar to DAD, with diffuse lower-lobe consolidation and GGOs; those with a mild/moderate course may have similar CT features of OP, characterized by both focal and diffuse lung abnormalities.

Our results differ from those of Dai JH et al. [25], where the most common pattern of AFOP was represented by lobar consolidation similar to OP; these differences may be related to the heterogeneity of two population samples for the prevalence of COVID-19 pneumonia in our patients.

GGO nodules were the most frequent CT features in AFOP patients, whereas pleural effusion and other atypical findings were uncommon; these data are in accordance with Chen H [3] and colleagues, who found that consolidations were more often present in idiopathic AFOP patients, while GGOs were more often present in secondary AFOP patients.

Interestingly, our study observed a predominance of female patients (70%) among AFOP cases, which contrasts with previous reports that generally suggest a male predominance in AFOP or no clear gender bias. This discrepancy may reflect population-specific factors, sampling variability, or potentially differing immunologic responses to SARS-CoV-2 between sexes. Emerging evidence [26] suggests that female patients may mount a more robust innate and adaptive immune response to viral infections, possibly influencing the inflammatory and reparative processes in the lung. The hormonal modulation of immune pathways may also contribute to a differential susceptibility to post-viral lung injury patterns, including AFOP. Further studies are needed to explore these mechanisms and validate the observed gender-related trend.

Our observations also demonstrate a strong positive correlation (*r* = 0.991, *p* < 0.05) between histological fibrin deposition and the main HRTC patterns of AFOP, including GGO nodules or pseudonodular consolidations and diffuse patchy GGOs with varying degrees of consolidation. This correlation underscores the diagnostic utility of imaging in assessing AFOP, emphasizing that areas of fibrin-rich exudate significantly contribute to lung parenchymal opacity. In the heightened inflammatory context of post-COVID-19, this interplay between histology and radiology could manifest more pronouncedly, necessitating multidisciplinary approaches to diagnosis and patient management.

Given AFOP’s frequent radiological mimicry of neoplasia, histopathological confirmation remains essential in avoiding misdiagnosis and unnecessary surgical interventions, especially in patients presenting with nodular pulmonary opacities and a history of COVID-19 [4,27]. Thus, clinical, radiological, and histopathological integration is critical for optimizing diagnostic accuracy, therapeutic strategies, and patient outcomes in AFOP.

## 5. Conclusions

In this study, we demonstrated a significant connection between AFOP and COVID-19 infection, highlighting that AFOP can be triggered by SARS-CoV-2. The observed association between ground-glass opacities (GGOs) and histopathological findings emphasizes the necessity for a multidisciplinary approach integrating imaging reports with histopathological evaluations to achieve accurate diagnosis. Clinicians and pathologists must remain vigilant when assessing nodular pulmonary opacities in post-COVID-19 patients given that AFOP’s radiological appearance can closely mimic malignancies, potentially leading to unnecessary surgical interventions. Additionally, the observed predominance of female patients and the upper-lobe distribution of AFOP nodules suggest distinctive pathogenic mechanisms that warrant further investigation. Moving forward, employing an integrated clinical, radiological, and histopathological strategy will be crucial in enhancing diagnostic precision, guiding therapeutic choices, and improving patient outcomes in AFOP, especially following viral lung injuries.

### Limitations

This study has several limitations. First, it is a retrospective analysis conducted at a single tertiary center, which may limit the generalizability of the findings. Second, the sample size, particularly for AFOP cases, is relatively small due to the rarity of this presentation, which could impact the statistical power of the comparisons. Third, there is a potential selection bias, as all included cases were solitary pulmonary nodules suspicious for malignancy that underwent surgical resection, inherently excluding patients with diffuse or multifocal lung disease. Finally, although radiologic–pathologic correlation was performed, the interpretation of imaging findings may be influenced by the clinical context and surgical indication, which could affect classification and comparison.

Despite these limitations, this study provides valuable insights into a distinct and underrecognized post-COVID-19 manifestation, emphasizing the importance of multidisciplinary diagnostic approaches.

## Figures and Tables

**Table 1:** Patient demographics and clinical presentations.

	AFOP	OP	*p*
**Total**	20	119	
**Female**	14	56	**
**Male**	6	63	**
**Mean Age**	65.85	66.83	Ns
**Smokers**	5.0	42.0	Ns
**Cannabinoid Users**	1.0	0.0	Ns
**E-Smokers**	3.0	1.0	Ns
**COVID-19 Cases**	11.0	1.0	***
**Other Pulmonary Infections**	0.0	14.0	*

* = *p* < 0.05; ** = *p* < 0.005; *** = *p* < 0.001; Ns = not significant.

## Data Availability

The data presented in this study are available upon request from the corresponding author. The data are not publicly available due to the privacy policies of the centers involved in the study.
